# Impact of Self‐Reported Long‐Term Mental Health Morbidity on Help‐Seeking and Diagnostic Testing for Bowel‐Related Cancer Symptoms: A Vignette Study

**DOI:** 10.1002/cam4.70426

**Published:** 2024-12-06

**Authors:** Flavia Pennisi, Giovanni Emanuele Ricciardi, Christian von Wagner, Lauren Smith, Aradhna Kaushal, Georgios Lyratzopoulos, Samuel William David Merriel, Willie Hamilton, Gary Abel, Jose Maria Valderas, Cristina Renzi

**Affiliations:** ^1^ PhD National Programme in One Health Approaches to Infectious Diseases and Life Science Research, Department of Public Health, Experimental and Forensic Medicine University of Pavia Pavia Italy; ^2^ School of Medicine Università Vita‐Salute San Raffaele Milano Italy; ^3^ Research Department of Behavioural Science and Health University College London London UK; ^4^ Centre for Primary Care & Health Services Research University of Manchester Manchester UK; ^5^ Department of Health and Community Sciences, Faculty of Health and Life Sciences University of Exeter Exeter UK; ^6^ Department of Family Medicine National University Health System Singapore City Singapore; ^7^ Centre for Research on Health Systems Performance National University of Singapore Singapore City Singapore

**Keywords:** cancer diagnosis, colorectal cancer, mental disorders, mental health

## Abstract

**Objective:**

To investigate if pre‐existing mental health morbidity (MHM) might influence help‐seeking and willingness to undergo diagnostic investigations for potential colorectal cancer (CRC) symptoms.

**Methods:**

An online vignette survey was completed by 1307 adults aged > 50 years recruited through Prolific, a UK panel provider. Participants self‐reported any chronic physical or MHM. After having been presented with vignettes describing new onset symptoms (rectal bleeding or change in bowel habit), participants answered questions on symptom attribution and attitudes to investigations. Using multivariable logistic regression we examined the association between MHM and symptom attribution, intended help‐seeking, and willingness to undergo investigations, controlling for socio‐demographic factors and physical morbidities.

**Results:**

Self‐reported MHM (reported by 14% of participants) was not associated with cancer symptom attribution (29% of participants with or without MHM mentioned cancer as a possible reason for rectal bleeding and 14% for change in bowel habit). Individuals with self‐reported MHM were less likely to contact a GP if experiencing a change in bowel habit (19% vs. 39%; adjusted (a)OR = 0.34, 95% CI 0.19–0.60) and to mention rectal bleeding to their GP (83% vs. 89%, aOR = 0.49, 95% CI 0.26–0.94). Although most participants would be willing to undergo a colonoscopy for these high‐risk symptoms, those with depression/anxiety were less willing (90% vs. 96%; aOR: 0.37, 95% CI 0.16–0.87).

**Conclusions:**

Individuals with self‐reported MHM are less likely to seek help and less willing to undergo investigations for high‐risk symptoms. Targeted support, for example, through additional mental health nurses, might facilitate prompt cancer diagnosis for the large group of people with MHM.

## 1 Background

Mental health morbidity (MHM) affects large proportions of the general population in Western countries, with a quarter of adults self‐reporting MHM and one in eight (13%) having a primary care record of anxiety or depression [1]. According to previous studies, individuals with MHM are at increased risk of advanced‐stage cancer diagnosis [2] and premature death, approximately 15–20 years earlier than the general population [3, 4, 5]. In 2018, the estimated 5‐year overall survival proportion was 0.66 (95% CI, 0.60–0.71) and 0.74 (95% CI, 0.72–0.76) for cancer patients with and without a preexisting mental disorder diagnosis, respectively [2].

In the UK, colorectal cancer (CRC) is diagnosed at an advanced stage or following an emergency presentation in 53% and 24% of cases, respectively [6]. Individuals with MHM might be particularly disadvantaged, possibly due to a lower uptake of cancer screening, delays before seeking help from a doctor, and prolonged time before undergoing diagnostic investigations for cancer symptoms [7]. A recent study on colon cancer patients visiting their doctor with high‐risk cancer symptoms reported less frequent endoscopy use in the 24 months pre‐cancer diagnosis for patients with MHM, more than 2‐fold longer diagnostic intervals for patients with versus without MHM, and 63% higher odds of emergency cancer diagnosis, independently of physical comorbidity, age, and socioeconomic deprivation [7].

**FIGURE 1:**
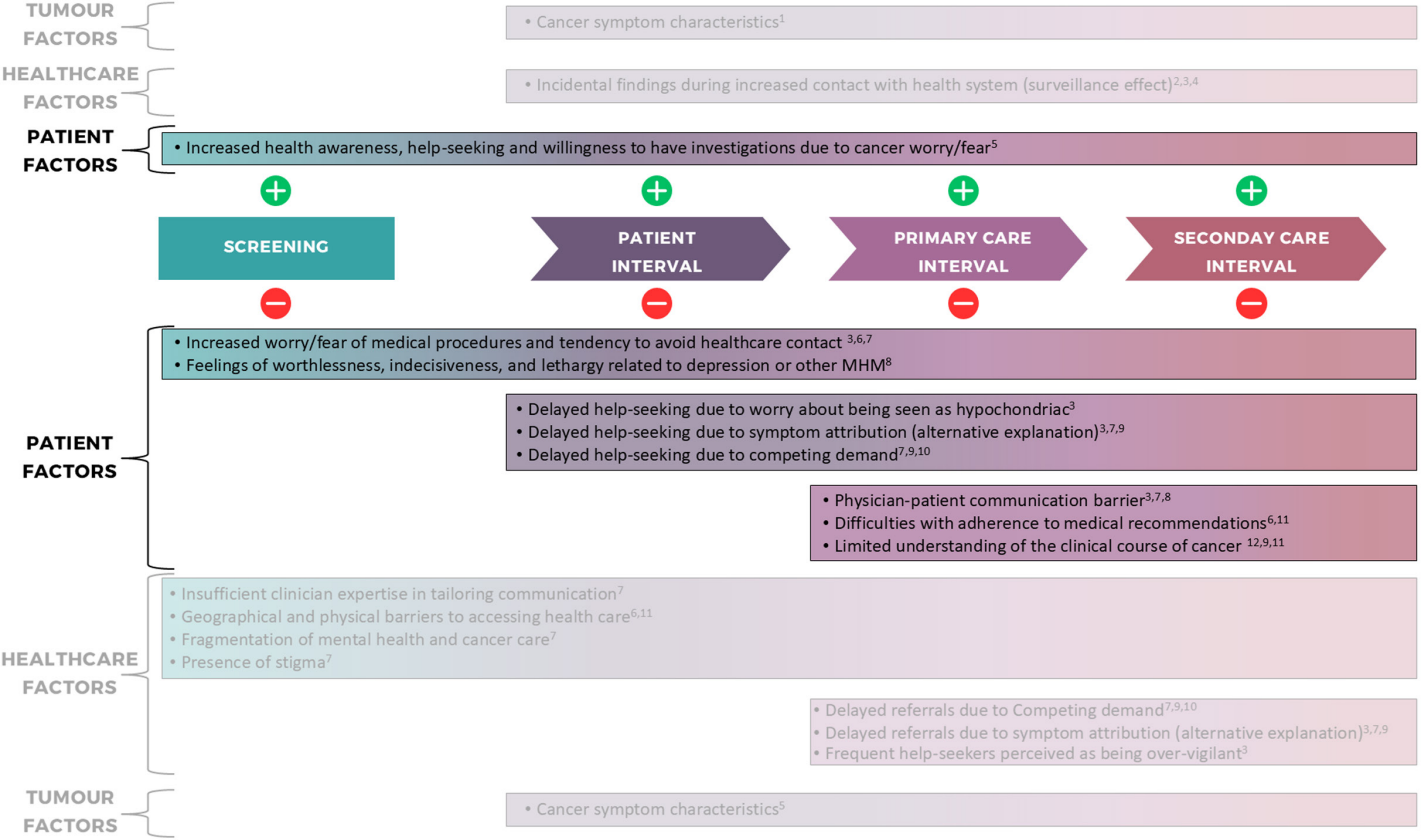
Patient, healthcare and tumor‐related factors that may influence diagnostic pathways and the timely diagnosis of cancer in patients with mental health morbidities. Cancer diagnosis can be positively or negatively influenced (green or red circles in the figure, respectively) by patient, healthcare‐, and tumor‐related factors. For example, patients with anxiety or depression could delay help‐seeking if attributing cancer symptoms to their mental health condition (alternative explanations), or if their mental health takes priority over investigating new symptoms (competing demands) [7, 12, 15, 16, 17, 18, 19, 20, 21, 22]. In contrast, frequent medical visits or tests performed for the mental health condition could lead to earlier cancer diagnosis (surveillance effect).

Limited evidence exists on the mechanism through which MHM can influence cancer diagnosis. Patients with MHM may face barriers in accessing healthcare, with disparities in cancer care likely resulting from interrelated issues at the patient, physician, and healthcare system levels, as well as being influenced by cancer type and symptoms [8] (Figure 1). In particular, patients with MHM might be less willing to undergo investigations or their mental health condition may provide an alternative explanation for possible cancer symptoms. For example, in the case of CRC symptoms, such as a change in bowel habit, patients and/or healthcare providers may attribute symptoms to the pre‐existing mental health condition (anxiety), rather than to an as‐yet undiagnosed cancer [9, 10]. Furthermore, fragmented healthcare services and difficulties in accessing healthcare (e.g., due to geographical barriers, and waiting time for seeing a doctor) can exacerbate disparities in care for MHM patients. Stigma has been suggested to play an important role, including the perception of oneself as stigmatized and social stigma and prejudice by others [10, 11]. In contrast, sometimes chronic conditions might facilitate, rather than hinder, the timely diagnosis of cancer, thanks to more frequent healthcare contacts [10, 12], leading to opportunities to diagnose cancer promptly through the surveillance mechanism [13, 14].

The study aimed to examine variations in symptom attribution, help‐seeking, and willingness to undergo diagnostic tests for potential CRC symptoms between individuals with and without MHM. We hypothesized that individuals with long‐term mental MHM are less likely to attribute CRC symptoms to cancer, less likely to seek medical assistance, and less willing to undergo diagnostic investigations, such as colonoscopy, compared to those without MHM.

## 2 Methods

### 2.1 Study Design

We gathered information on the role of MHM in influencing attitudes on diagnostic investigations for possible CRC symptoms via a vignette survey. To that aim, we developed two vignettes describing new‐onset symptoms of rectal bleeding or change in bowel habits. Vignettes are short hypothetical scenarios representing real‐life situations [23]. They are used in diagnostic research as they allow for the manipulation of symptoms and clinical presentations in real‐life situations while keeping the context constant [9, 10], to examine reactions and intended behaviors [12]. Participants were recruited in August 2021 through Prolific (www.prolific.co), a well‐established online platform designed for academic research recruitment. Prolific provides access to a large, diverse pool of participants who are fully informed about their involvement in scientific studies, which helps ensure high engagement and reliable data [24]. The platform is widely utilized across disciplines, such as psychology [25] economics [26], and health sciences [27], and has been validated through numerous peer‐reviewed studies. At the time of this study, Prolific had over 5500 UK‐based participants aged 50 and above, making it an ideal platform for identifying individuals who met the study's demographic and eligibility requirements. Quota sampling was implemented to ensure the recruitment of participants met predetermined demographic targets, including age and gender distributions, in alignment with the study's eligibility criteria. Eligible participants were contacted via email and invited to take part in the study, which involved reading a vignette related to symptom perception and help‐seeking. All participants examined in this study were exposed to both vignettes 1 and 2. The word ‘cancer’ was not mentioned to the study participants to mask the study aim and to reduce priming and response bias, similar to previous studies. After reading the vignettes, participants were asked pre‐coded and open questions about symptom attribution, intended help‐seeking, and attitudes toward investigations [28]. Participants were offered a £1.25 incentive upon survey completion, based on the standard compensation of £5 per hour provided by Prolific for approximately 15 min. This study was part of a wider project using online vignette surveys to investigate the role of pre‐existing morbidities in influencing the diagnosis of cancer, in which information on multiple morbidities, including MHM, was gathered. They were also asked about the number of annual GP visits before and during the pandemic. The overall project methods have been previously described [29].

### 2.2 Study Participants

Participants were recruited in August 2021 through Prolific, a survey provider. They were contacted by email to provide written informed consent. To be eligible, participants had to meet the following criteria: be 50 or older, live in the UK, and not have received a cancer diagnosis within the past 5 years. Prolific had approximately 5500 UK participants aged 50 or older during the study period. Eligible participants were contacted via email and invited to take part.

### 2.3 Vignettes and Questionnaire

We developed vignettes describing CRC symptoms considering that changes in bowel habits and rectal bleeding in people aged 50 or older warrant urgent referral for suspected cancer according to NICE guidelines [30]. Vignettes and questionnaires were developed with input from patient representatives, healthcare professionals, and researchers. To ensure that the final study material was tailored to participants, cognitive interviews with 22 individuals and a pilot study with 200 individuals were performed. The two resulting vignettes were:

- Vignette 1—rectal bleeding: “When you use the bathroom, you notice blood in your poo (rectal bleeding). Other than this symptom, you have noticed no other changes.”
- Vignette 2—change in bowel habit: “You notice you have had changes in your normal bowel habit (such as looser poo, pooing more often or constipation). Other than this symptom, you have noticed no other changes.”

### 2.4 Study Variables

#### 2.4.1 Main Outcomes

##### 2.4.1.1 Symptom Attribution

Participants were asked to write in a free‐text format any potential cause they believed could be responsible for their symptoms. We employed content analysis to categorize the answers on attribution to cancer, benign bowel disease, hemorrhoids, anal tear/fissure, constipation, dietary changes or food poisoning, and medication.

##### 2.4.1.2 Intended Help‐Seeking

Participants chose between 13 pre‐coded options, such as “Talk to family members” or “Contact the GP” (complete list in Appendix 1, Table A3), which were presented in random order. They selected the likelihood of performing each action. A free‐text option was also given if they selected “Other.” Responses were dichotomized, with those indicating “probably would” or “Definitely would” categorized as “Would take action,” and those indicating “Probably wouldn't” or “Definitely wouldn't” categorized as “Wouldn't take action.” In this study, we analyzed only the following actions: “Contact the GP,” “Mention if you saw the GP for another reason,” “Contact a nurse,” “Mention if you saw a nurse for another reason,” “Dismiss as something not to worry about.”

##### 2.4.1.3 Willingness/Attitudes to Undergo Diagnostic Investigations

Participants were asked if they would be willing to have a colonoscopy/sigmoidoscopy after reading vignette 1 (rectal bleeding), and if they would be willing to have a stool test after reading vignette 2. Possible answers were “yes” or “no.” In cases of a negative response, the reason was explored with an open‐ended question. Participants were also asked if they had ever undergone a stool test or colonoscopy/sigmoidoscopy in the past, with responses being “no,” “yes for screening,” and “yes for symptoms.”

Additional details regarding the development of the vignettes can be found in our previously published paper [28].

#### 2.4.2 Explanatory Variables

##### 2.4.2.1 Self‐Reported MHM

The question on chronic conditions was adapted from the GP Patient Survey [31], asking participants “We would like to know about any health problems you may have.” Participants were invited to select any of the 21 pre‐coded conditions, plus an open‐ended option. Participants reporting “long‐term mental health problems (e.g., depression, anxiety)” were classified as having a pre‐existing MHM.

##### 2.4.2.2 Self‐Reported Other Physical Comorbidities

Self‐reported socio‐demographic characteristics. Information on age, gender, ethnicity, and educational level was also collected.

Additionally, past fecal occult blood test or colonoscopy/sigmoidoscopy information was collected by asking participants: “Have you ever had a stool sample?” and “Have you ever had a colonoscopy/sigmoidoscopy?”, with pre‐coded answers: “no,” “yes, for screening,” and “yes, for symptoms.”

### 2.5 Analysis

We employed content analysis to categorize the answers on attribution to cancer and benign gastrointestinal (GI) conditions, including hemorrhoids, constipation dietary changes, or food poisoning. We employed Chi‐squared tests to assess the differences in participant characteristics between those with and without MHM. Multivariable logistic regression was used to analyze the association between MHM and the following outcomes: symptom attribution, intended help‐seeking, and willingness to undergo diagnostic investigations. Each outcome was evaluated in a separate multivariable model, for a total of 24 multivariable logistic regressions, which accounted for potential confounding factors, including age, gender, ethnicity, previous diagnostic testing (stool and colonoscopy), and a total number of selected physical chronic conditions (including cardiovascular, respiratory conditions, diabetes, and others), in line with previous research and clinical reasoning. We used 2‐sided tests and considered *p* < 0.05 as statistically significant. We employed Stata statistical software version 17 (StataCorp) for the analyses.

## 3 Results

### 3.1 Participant Characteristics

A total of 1456 participants initially took part in the study. After excluding 108 individuals with incomplete responses and 59 with cancer in the last 5 years, 1287 participants remained. Among them, 61% were female, and 87% were of white ethnic background, which aligns with Prolific's participant characteristics. About 14% (*n* = 183) reported having a mental health condition. Those with MHM tended to be younger, more often of white ethnicity, and had a significantly higher prevalence of two or more additional chronic conditions compared to those without MHM (66% vs. 43%) (Table 1).

**TABLE 1:** Characteristics of participants with and without Mental Health Morbidity (MHM).

	Total	With MHM	Without MHM	*p*
	*N* = 1287	*N* = 183	*N* = 1104	
Age				
50–59	791 (61.5%)	133 (72.7%)	658 (59.6%)	0.001
60–69	399 (31.0%)	45 (24.6%)	354 (32.1%)	
70+	97 (7.5%)	5 (2.7%)	92 (8.3%)	
Gender				
Male	500 (38.9)	78 (42.6%)	422 (38.2%)	0.122
Female	782 (60.8%)	103 (56.3%)	679 (61.5%)	
Prefer not to say^a^	5 (0.4%)	2 (1.1%)	3 (0.3%)	
Ethnic group				
White	1123 (87.3%)	172 (94.0%)	951 (86.1%)	0.003
Other	164 (12.7%)	11 (6.0%)	153 (13.9%)	
Other comorbidities				
0	319 (24.8%)	22 (12.0%)	297 (26.9%)	< 0.001
1	377 (29.3%)	41 (22.4%)	336 (30.4%)	
2+	591 (45.9%)	120 (65.6%)	471 (42.7%)	
Past stool test				
Yes, for screening	475 (36.9%)	58 (31.7%)	417 (37.8%)	0.012
Yes, for symptoms	226 (17.6%)	46 (25.1%)	180 (16.3%)	
No	586 (45.5%)	79 (43.2%)	507 (45.9%)	
Past colonoscopy/sigmoidoscopy				
Yes, for screening	101 (7.9%)	9 (4.9%)	92 (8.3%)	< 0.001
Yes, for symptoms	260 (20.2%)	56 (30.6%)	204 (18.5%)	
No	926 (72.0%)	118 (64.5%)	808 (73.2%)	
GP visits pre‐pandemic				
0	445 (34.6%)	35 (19.1%)	410 (37.1%)	< 0.001
1	321 (24.9%)	37 (20.2%)	284 (25.7%)	
2–9	499 (38.8%)	103 (56.3%)	396 (35.9%)	
10+	22 (1.7%)	8 (4.4%)	14 (1.3%)	
GP visits during pandemic				
0	370 (28.8%)	24 (13.1%)	346 (31.3%)	< 0.001
1	278 (21.6%)	36 (19.7%)	242 (21.9%)	
2–9	610 (47.4%)	116 (63.4%)	494 (44.8%)	
10+	29 (2.3%)	7 (3.8%)	22 (2.0%)	

^a^ Group removed from further analyses due to small numbers.

Overall, 28% of participants had undergone a colonoscopy/sigmoidoscopy in the past, while more than half (55%) had a history of stool tests, regardless of MHM status. However, MHM respondents had a lower proportion of past stool tests for screening but more for symptoms than those without MHM (Table 1). Additionally, a smaller percentage of MHM respondents reported past colonoscopy/sigmoidoscopy for screening (5% vs. 8%) but a higher percentage for symptoms (31% vs. 19%) compared to non‐MHM respondents.

The frequency of GP visits was higher among those with MHM, with an increase observed both pre‐pandemic and post‐pandemic for both groups. Before the pandemic, 56% of MHM participants and 36% of non‐MHM participants had 2–9 GP visits annually, while following March 2020, these percentages rose to 63% and 45%, respectively, with fewer participants reporting 0–1 GP visits per year.

### 3.2 Symptom Attribution

Overall, following the vignette presentation, participants most commonly attributed rectal bleeding to hemorrhoids (30%) or cancer (29%), while the change in bowel habit was often attributed to dietary changes by both MHM and non‐MHM participants (36% vs. 33%, respectively), followed by cancer attribution (15% and 14%, respectively). There were no significant differences in symptom attribution between participants with and without MHM for either vignette. The multivariable logistic regressions demonstrated that there was no significant association between MHM status and the attribution of cancer symptoms, even after accounting for potential confounding variables (data shown in Appendix 1, Tables A1 and A2).

### 3.3 Intended Help‐Seeking

**FIGURE 2:**
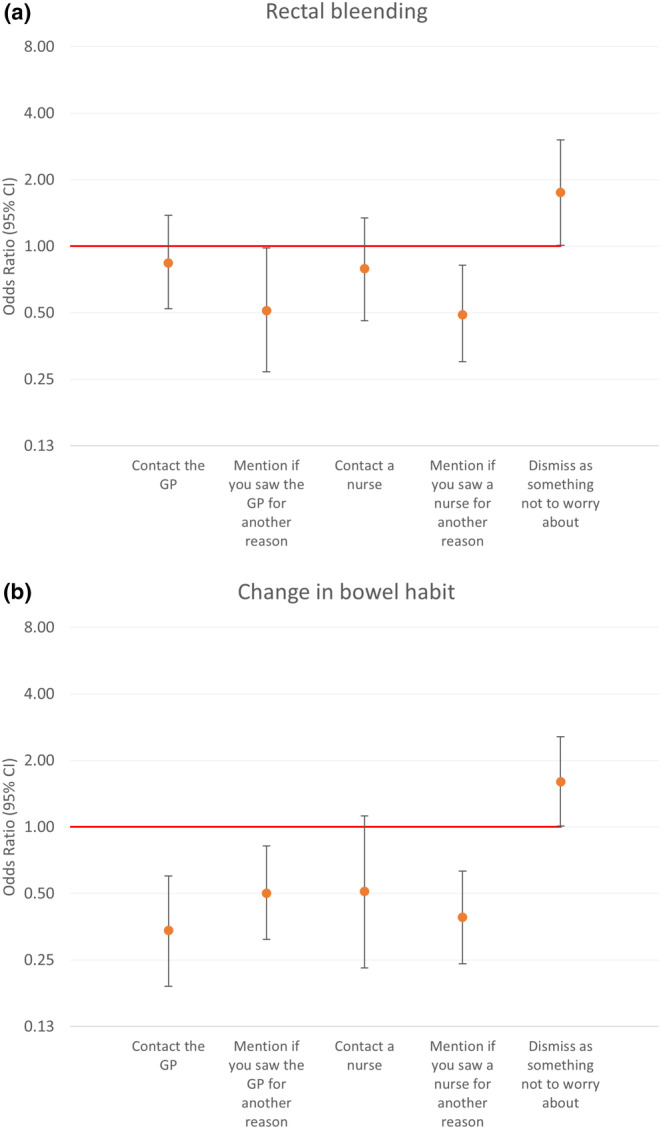
(a) Intended help‐seeking reported by participants with mental health morbidity versus those without when experiencing rectal bleeding (Vignette 1): Multivariable logistic regression odds ratios, adjusted for age, gender, ethnicity, comorbidity number and previous investigations. (b) Intended help‐seeking reported by participants with mental health morbidity versus those without when experiencing a change in bowel habit (Vignette 2): Multivariable logistic regression odds ratios, adjusted for age, gender, ethnicity, comorbidity number and previous investigations.

For rectal bleeding (vignette 1), participants with MHM most commonly sought online information (88%). At the same time, those without MHM typically mentioned it to their GP during another visit, while MHM individuals were less likely to mention it (83% vs. 89%; adjusted OR: 0.51, 95% CI: 0.27–0.98) or nurse consultations (65% vs. 77%; adjusted OR: 0.49, 95% CI: 0.30–0.82) for other reasons. They were also more inclined to dismiss rectal bleeding as non‐concerning compared to non‐MHM participants (26% vs. 18%; adjusted OR: 1.75, 95% CI: 1.01–3.02) (Figure 2). Moreover, participants with MHM were less inclined to contact the GP compared to non‐MHM participants (83% vs. 89%; adjusted OR: 0.51, 95% CI: 0.27–0.98).

For the change in bowel habit (vignette 2), both MHM and non‐MHM participants frequently opted to “wait and see what happens,” with a higher proportion among those with MHM (92% vs. 87%), although this difference was not statistically significant. There were significant differences based on MHM status in seeking help from a GP or nurse for this symptom. Participants with MHM were less likely to contact the GP (19% vs. 39%; adjusted OR = 0.34, 95% CI: 0.19–0.60), mention the symptom during GP visits for other reasons (61% vs. 75%; adjusted OR = 0.50, 95% CI: 0.31–0.82), or mention it during nurse visits for different reasons (43% vs. 62%; adjusted OR: 0.39, 95% CI: 0.24–0.63) (Figure 2b). Moreover, multivariable models showed a ower likelihood of help‐seeking for individuals who had previously undergone a colonoscopy.

### 3.4 Willingness to Undergo Diagnostic Investigations for New Symptoms

Overall, 95% of participants were willing to undergo colonoscopy/sigmoidoscopy for rectal bleeding, and 98% were willing to have a stool test for new change in bowel habit. MHM status did not significantly affect willingness to undergo stool testing for new symptoms. However, those who had previously undergone a stool test and individuals aged 60–69 were less willing to repeat the test for new symptoms. Participants with MHM were significantly less willing than those without to undergo colonoscopy/sigmoidoscopy for new CRC symptoms like change in bowel habit or rectal bleeding, even though it is the majority (90% versus 96%; adjusted OR: 0.37, 95% CI: 0.16–0.87) (Table 2).

**TABLE 2:** Willingness to undergo diagnostic testing for new symptoms (rectal bleeding or change in bowel habit) by participants with MHM versus those without: Multivariable logistic regression odds ratios, adjusted for gender, age, ethnic group, comorbidity number and previous testing history.

	Stool sample		Colonscopy	
	OR (95% CI)	*p*	OR (95% CI)	*p*
Unadjusted model				
MHM				
No	1.0		1.0	
Yes	0.45 (0.12–1.71)	0.240	0.45 (0.20–0.99)	0.048
Adjusted model				
MHM				
No	1.0		1.0	
Yes	0.27 (0.61–1.19)	0.105	0.38 (0.16–0.90)	0.023
Gender				
Men	1.0		1.0	
Women	1.60 (0.47–5.46)	0.425	0.56 (0.26–1.21)	0.147
Age				
50–59	1.0		1.0	
60–69	0.16 (0.04–0.67)	0.014	0.60 (0.26–1.38)	0.242
70+	0.19 (0.02–1.94)	0.170	0.77 (0.16–3.61)	0.718
Comorbidities				
0	1.0		1.0	
1	0.45 (0.10–2.06)	0.549	0.63 (0.24–1.70)	0.328
2+	1.57 (0.28–8.84)	0.693	0.73 (0.28–1.90)	0.765
Ethnic groups				
No white	1.0		1.0	
White	0.65 (0.12–3.53)	0.685	0.82 (0.32–2.11)	0.692
Previous colonoscopy				
No	1.0		1.0	
Yes (for screening or symptoms)	1.61 (0.58–4.52)	0.348	0.42 (0.17–1.02)	0.062
Previous stool test				
No	1.0		1.0	
Yes (for screening or symptoms)	0.26 (0.09–0.69)	0.006	0.82 (0.53–1.26)	0.374

Among those unwilling to have a stool test, the main reasons were perceived as unnecessary (40%) and embarrassment (30%). Similarly, embarrassment and feelings of anxiety or nervousness were the most common reasons cited by participants unwilling to undergo colonoscopy/sigmoidoscopy (34%). Other reasons included discomfort (16%), invasiveness (13%), or preferring alternative tests first (13%).

## 4 Discussion

### 4.1 Main Findings and Comparison With the Literature

The study found that participants with self‐reported long‐term MHM were significantly less likely to contact their GP for high‐risk cancer symptoms, like change in bowel habits or rectal bleeding, compared to those without MHM. They were also less likely to mention these symptoms during medical encounters performed for other reasons. Notably, for the change in bowel habits, both MHM and non‐MHM participants commonly chose to “wait and see what happens,” with a higher proportion observed among those with MHM. However, this difference was not statistically significant. Additionally, while most participants with or without MHM were willing to undergo investigations for possible CRC symptoms, those with MHM reported a lower propensity to have a colonoscopy, often due to feelings of embarrassment, anxiety, or fear.

The link between mental health and timely cancer diagnosis is poorly understood. We lack insight into how MHM might affect symptom interpretation, help‐seeking behavior, and attitudes toward diagnostic tests. Our study found no difference in cancer attribution based on MHM status, contradicting assumptions that MHM increases the risk of misinterpreting potential cancer symptoms [32, 33]. Some studies propose that symptoms like change in bowel habit or abdominal pain in individuals with MHM may be attributed to anxiety disorder [34], medication side effects [12], or irritable bowel syndrome. While our study was not specifically designed to examine a large variety of symptoms, the findings suggest that MHM per se does not affect cancer attribution, despite MHM individuals might experience some symptoms more frequently due to benign causes. Overall, 15% of participants, regardless of MHM status, considered cancer as a potential cause for change in bowel habit, aligning with general population surveys on cancer awareness [35]. Despite opportunities for increased awareness, individuals with MHM encounter specific barriers in translating cancer awareness into health behaviors, such as seeking help from GPs or accepting diagnostic tests for high‐risk symptoms.

In line with the competing demands mechanism [22], hesitancy to seek help might be related to patients with MHM having other more urgent needs that take priority. In our study, participants with MHM reported a lower likelihood of seeking help from the GP or nurse and mentioning CRC symptoms when seeing them for other reasons. This could be related to patients prioritizing discussing MHM or other health issues rather than abdominal symptoms, given the limited time of primary care consultation [36, 37].

Another explanation is that a significant portion of MHM individuals in our study (30%) had undergone past colonoscopies. This could have reassured them, leading to reduced worry and less inclination to seek help for abdominal symptoms. Consistent with the surveillance mechanism [13], individuals with chronic conditions often have more frequent healthcare interactions, offering increased chances to report potential cancer symptoms. Possible reasons for not reporting symptoms may include fear of cancer, lower self‐efficacy, and candidacy assessment, whereby individuals evaluate their eligibility for healthcare access and legitimize their engagement with services [38]. Although our findings indicate that individuals with MHM had higher annual GP visit frequencies compared to those without, this didn't translate into a higher likelihood of reporting CRC high‐risk symptoms.

Patients with MHM were less inclined to undergo colonoscopy when experiencing CRC high‐risk symptoms, even after accounting for previous colonoscopies or stool tests, as well as socio‐demographic factors and other comorbidities. Research has demonstrated that up to 45.2% of eligible individuals for colonoscopy experience emotional and cognitive symptoms linked to cancer anxiety, a significant barrier to participation [39]. Additionally, colonoscopy is often seen as invasive, uncomfortable, and embarrassing [40]. Our study suggests that MHM patients may have heightened fears regarding invasive procedures like colonoscopies. This underscores how MHM not only deters seeking medical help but also affects readiness for vital diagnostic procedures, potentially contributing to diagnostic delays and advanced‐stage cancer diagnoses [2, 18, 41] despite increased GP consultations. Even though seeking medical help more often due to lower symptom tolerance, this does not offset reduced willingness for testing, as reflected in decreased screening participation. MHM is associated with both lower screening participation and heightened anxiety regarding colonoscopies, further complicating timely diagnosis efforts [39, 40].

Addressing these concerns and providing appropriate support and reassurance to people with MHM with symptoms for which further investigations are warranted are critical steps in improving compliance with recommended investigations for CRC and may contribute to decreasing inequalities in cancer survival.

### 4.2 Strengths and Limitations

The study's strengths included the use of online vignettes, a methodology extensively used in diagnostic research for elucidating cognitive and attitudinal drivers of behavior. Additionally, the inclusion of open‐ended questions participants could provide insights into their perspective on sensitive issues, such as reasons for not being willing to undergo investigations. The high survey completion rate (88%) supports the robustness of the findings.

The study has limitations. While helpful in examining responses in controlled scenarios, Vignettes may not fully reflect real‐world behaviors and attitudes. Simulated symptoms lack the depth of real‐life experiences, potentially influencing participants' responses. Vignettes primarily assess intended rather than actual behavior, although forming intentions is an important step toward action [42, 43]. This study analyzed data collected as part of a wider project focusing primarily on diabetes and the diagnosis of CRC. While data was also collected on other conditions, including MHM, the available information only allowed for partial elucidation of the complexities in health attitudes and behaviors specific to people with MHM. In addition, the number of participants with MHM was relatively small, and with limited representation of ethnic minorities, which was however in line with the UK population data [44]. Finally, the broad nature of our MHM question restricted our ability to explore specific types of MHM in depth.

Moreover, one limitation that should be acknowledged is the large number of analyses completed, which increases the risk of Type 1 error. The multiple comparisons made in the study may have inflated the likelihood of identifying significant associations by chance, and this risk should be taken into account when interpreting the results.

Another relevant limitation of this study is that the diagnosis of MHM is based on self‐reported data rather than on the use of validated diagnostic scales. Although self‐reported measures are a common feature of population‐based studies, they lack the precision of more robust diagnostic tools, such as structured clinical interviews or validated questionnaires specifically designed to assess MHM [45, 46, 47, 48, 49]. In this study, participants were asked to indicate whether they had long‐term MHM, such as anxiety or depression, without the use of standardized mental health assessments. Such variability in the interpretation and reporting of mental health status may lead to misclassification or underreporting. Consequently, the utilization of self‐reported MHM may compromise the precision of the findings, and this should be considered when interpreting the results. It would be beneficial for future research to incorporate validated measures to ensure more accurate identification of mental health conditions and their impact on help‐seeking behavior and diagnostic testing.

Quota sampling may have introduced selection bias, potentially affecting the representativeness of the sample. This method, while useful for ensuring a balanced demographic composition, may not fully reflect the broader population, limiting the generalizability of the findings. Future studies should consider using probabilistic sampling methods to minimize this bias.

### 4.3 Clinical Implications

Further research with more extensive and diverse samples of individuals with MHM is essential to understand why they are less likely to seek help from healthcare professionals when experiencing possible cancer symptoms. Investigating factors like fear, access barriers, and stigma related to MHM could offer valuable insights for interventions. Qualitative research could delve into reasons for reluctance to undergo colonoscopies for cancer symptoms, including the impact of previous negative diagnostic experiences. We need additional studies to develop effective approaches for promoting timely cancer diagnosis among individuals with MHM, emphasizing holistic healthcare that addresses both physical and mental well‐being. Addressing misconceptions about symptoms and testing is crucial, with tailored advice and educational materials for individuals with MHM. Healthcare providers should explore strategies to reduce barriers to colonoscopies, such as clear information, sedation options, and support for managing embarrassment and fear. Mental health nurses, counseling, and peer support groups could offer additional assistance tailored to the needs and concerns of individuals with MHM.

### 4.4 Conclusions

In conclusion, the study has highlighted that patients with long‐term MHM have a lower propensity to seek help and to undergo investigations when experiencing high‐risk cancer symptoms, while these are essential steps for diagnosing cancer early. Targeted support for patients and healthcare providers might be necessary to overcome barriers and facilitate prompt cancer diagnosis for the large group of people with MHM.

## Author Contributions

**Flavia Pennisi:** conceptualization (equal), data curation (equal), formal analysis (lead), methodology (equal), resources (equal), software (equal), validation (equal), writing – original draft (lead), writing – review and editing (equal). **Giovanni Emanuele Ricciardi:** conceptualization (equal), data curation (lead), formal analysis (equal), investigation (equal), methodology (equal), software (equal), writing – original draft (equal). **Christian von Wagner:** conceptualization (equal), methodology (supporting), supervision (equal), writing – review and editing (lead). **Lauren Smith:** conceptualization (equal), methodology (equal), writing – review and editing (equal). **Aradhna Kaushal:** funding acquisition (equal), methodology (equal), supervision (equal), writing – review and editing (equal). **Georgios Lyratzopoulos:** conceptualization (equal), funding acquisition (equal), project administration (equal), supervision (equal), writing – review and editing (equal). **Samuel William David Merriel:** conceptualization (equal), funding acquisition (equal), methodology (supporting), resources (equal), writing – review and editing (lead). **Willie Hamilton:** data curation (supporting), formal analysis (supporting), methodology (equal), writing – review and editing (equal). **Gary Abel:** data curation (supporting), methodology (supporting), supervision (equal), writing – review and editing (equal). **Jose Maria Valderas:** data curation (equal), investigation (equal), validation (equal), writing – review and editing (equal). **Cristina Renzi:** conceptualization (lead), data curation (equal), funding acquisition (equal), methodology (equal), project administration (equal), supervision (lead), writing – review and editing (equal).

## Ethics Statement

Ethical approval was granted by the University College London Ethics Committee (N14687/006).

## Conflicts of Interest

The authors declare no conflicts of interest.

## Data Availability

The data that support the findings of this study are available on request from the corresponding author. The data are not publicly available due to privacy or ethical restrictions.

## Appendix 1

**TABLE A1:** Symptom attribution by participants with mental health morbidity versus those without when experiencing rectal bleeding (vignette 1): Multivariable logistic regression odds ratios, adjusted for gender, age, ethnic group, comorbidity number and previous testing history.

	MH problems (adjusted) OR (95% CI)	*p*
Cancer		
Yes	1.09 (0.77–1.55)	0.633
No	1.0	
Bowel disease		
Yes	0.71 (0.33–1.56)	0.400
No	1.0	
Hemorrhoids/Piles		
Yes	1.00 (0.70–1.43)	0.988
No	1.0	
Anal tear/fissure		
Yes	1.05 (0.58–1.91)	0.863
No	1.0	
Don't know		
Yes	1.05 (0.45–2.44)	0.905
No	1.0	
Constipation/straining		
Yes	0.86 (0.44–1.68)	0.654
No	1.0	

**TABLE A2:** Symptom attribution by participants with mental health morbidity versus those without when experiencing a change in bowel habit (vignette 2): Multivariable logistic regression odds ratios, adjusted for gender, age, ethnic group, comorbidity number and previous testing history.

	MH problems (adjusted) OR (95% CI)	*p*
Cancer		
Yes	1.11 (0.70–1.76)	0.646
No	1.0	
Bowel disease		
Yes	0.96 (0.58–1.58)	0.865
No	1.0	
Dietary changes		
Yes	1.22 (0.87–1.71)	0.254
No	1.0	
Stomach infection/food poisoning		
Yes	1.21 (0.72–2.03)	0.484
No	1.0	
Don't know		
Yes	0.76 (0.31–1.83)	0.535
No	1.0	
Caused by medication		
Yes	0.87 (0.39–1.95)	0.737
No	1.0	

**TABLE A3:** Complete list of pre‐coded actions people would take.

	Definitely would	Probably would	Probably wouldn't	Definitely wouldn't	Not applicable
Talk to members of your family about the symptoms?	☐	☐	☐	☐	☐
Go to the pharmacy (chemist) for advice	☐	☐	☐	☐	☐
Contact the GP about the change in [symptom]	☐	☐	☐	☐	☐
Mention the symptoms if you saw the GP for another reason	☐	☐	☐	☐	☐
Go to A&E	☐	☐	☐	☐	☐
Look up information about the symptoms online	☐	☐	☐	☐	☐
Wait and see what happens (e.g., if the symptoms get worse)	☐	☐	☐	☐	☐
Dismiss the symptoms as something not to worry about	☐	☐	☐	☐	☐
Contact a nurse about the symptoms	☐	☐	☐	☐	☐
Mention the symptoms if you saw a nurse for another reason	☐	☐	☐	☐	☐
Contact a diabetes specialist	☐	☐	☐	☐	☐
Contact an endocrinologist	☐	☐	☐	☐	☐
Other	☐	☐	☐	☐	☐
If ‘other’ please specify					
